# Transforming Toxicity into Therapy: Exploring Bilirubin’s Benefits and Its Molecular Role in Cardiac Health and Disease

**DOI:** 10.3390/biom16050625

**Published:** 2026-04-23

**Authors:** Michael I. Adenawoola, Zachary A. Kipp, Terry D. Hinds, David E. Stec

**Affiliations:** 1 Department of Physiology & Biophysics, Cardiovascular-Renal Research Center, Cardiorenal and Metabolic Diseases Research Center, University of Mississippi Medical Center, Jackson, MS 39216, USA; madenawoola@umc.edu; 2 Drug & Disease Discovery D3 Research Center, Department of Pharmacology and Nutritional Sciences, University of Kentucky College of Medicine, Lexington, KY 40506, USA; zachary.kipp@uky.edu (Z.A.K.); terry.hinds@uky.edu (T.D.H.J.)

**Keywords:** heme oxygenase, heart failure, cardiac metabolism, PPARα, urobilin

## Abstract

Bilirubin, historically recognized solely as a waste product of heme catabolism, has recently gained attention for its potential protective role in the cardiovascular system. Experimental and clinical studies suggest that bilirubin exhibits potent antioxidant, anti-inflammatory, anti-apoptotic, and cytoprotective properties that may protect the heart against oxidative stress, ischemia–reperfusion injury, and the progression of cardiovascular diseases, such as heart failure. As an endogenous hormone, bilirubin activates peroxisome proliferator-activated receptor-α (PPARα), a nuclear receptor that controls energy balance and lipid metabolism. Moderately elevated circulating bilirubin levels have been associated with a reduced risk of coronary artery disease, heart failure, and myocardial infarction; however, the mechanisms underlying bilirubin’s protective effects remain incompletely understood. Conversely, the gut microbiota’s metabolism of bilirubin to urobilin is detrimental, given urobilin’s association with cardiometabolic dysfunction. The therapeutic potential of bilirubin in the management of cardiovascular disease is becoming increasingly apparent, supported by preclinical research and emerging technologies that enhance bilirubin delivery via nanoparticles and methods to elevate plasma bilirubin levels. Collectively, these scientific advancements position bilirubin as a promising, biologically plausible endogenous therapeutic for the prevention and treatment of heart disease.

## 1. Introduction

With an estimated 18 million deaths per year, cardiovascular disease (CVD) continues to be the world’s leading cause of mortality [1]. In the last few decades, numerous major pharmacological and interventional therapies and advances, such as statins, antihypertensives, and percutaneous coronary procedures, have been introduced; however, patients continue to experience poor cardiovascular outcomes [2,3]. Instead of focusing on the underlying metabolic, oxidative, and inflammatory processes that cause heart damage and remodeling observed in many CVDs, most current treatments primarily target risk factors, such as elevated cholesterol levels, which are addressed by statins [4,5]. There is a need to explore new endogenous and exogenous compounds that can modulate cardiovascular function to address this therapeutic gap.

**Figure 1:**
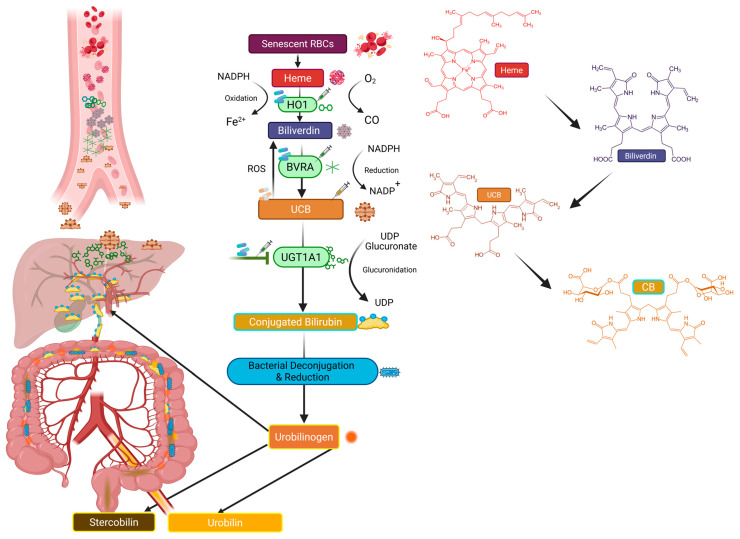
Pathway of bilirubin and urobilin generation. Plasma bilirubin levels are derived from the breakdown of red blood cells in the spleen through the action of heme oxygenase-1 (HO1) and biliverdin reductase A (BVRA). Unconjugated bilirubin is then conjugated in the liver by the enzyme uridine diphosphate glucuronosyltransferase 1A1 (UGT1A1). Conjugated bilirubin is deconjugated and then reduced in the intestine by the enzyme bilirubin reductase produced by gut bacteria to form urobilinogen. Urobilinogen is then rapidly oxidized to form urobilin, which is reabsorbed or further metabolized to stercobilin. Potential therapeutics can be developed by targeting HO1, BVRA, and UGT1A1. Created in Biorender. Michael Adenawoola (2026) https://app.biorender.com/illustrations/69de5c75c02bcd27f1f85691?slideId=032922eb-df34-4ed0-80d9-bf9d8ac070b6 (accessed on 14 April 2026).

Traditionally, bilirubin has been considered a toxic byproduct of heme catabolism, responsible for jaundice and, when severe, neurotoxicity in newborns at levels well-above the normal physiological range [6]. However, emerging evidence has since redefined bilirubin as a signaling molecule, an endogenous antioxidant, an anti-inflammatory, and a cytoprotectant [7,8,9]. The most intriguing function of bilirubin is its role as a hormone-signaling molecule, binding to and activating peroxisome proliferator-activated receptor alpha (PPARα) to regulate its transcriptional activity [10]. Generated through the enzymatic degradation of heme by heme oxygenase-1 (HO-1) and biliverdin reductase, as shown in Figure 1, bilirubin acts as a physiological regulator of redox balance and inflammation [11]. Epidemiological studies have demonstrated an inverse relationship between serum bilirubin levels and CVD risk. Individuals exhibiting mild hyperbilirubinemia (such as those with Gilbert’s syndrome) show reduced incidence of atherosclerosis, coronary artery disease, and myocardial infarction without liver dysfunction or toxicity [12,13]. At the mechanistic level, bilirubin exerts cardioprotective effects through multiple pathways. It can stimulate cardiac fatty acid metabolism by activating PPARα. It scavenges reactive oxygen and nitrogen species, inhibits vascular smooth muscle cell proliferation, and modulates endothelial nitric oxide synthase (eNOS) activity [14,15]. Moreover, bilirubin attenuates mitochondrial dysfunction, reduces lipid peroxidation, and preserves cardiac contractility under stress conditions, such as ischemia–reperfusion injury [16,17]. These multifaceted actions suggest that bilirubin may act on several levels to maintain cardiac homeostasis.

This review explores how bilirubin influences heart health and disease, emphasizing its roles in myocardial energy metabolism, vascular function, and redox signaling. It also reviews preclinical and clinical findings that support bilirubin’s potential as a therapy for cardiovascular conditions and discusses future opportunities to use this natural molecule in cardioprotective strategies.

## 2. Bilirubin Formation and Metabolism

### 2.1. Bilirubin Pools and Plasma Dynamics

Unconjugated (indirect) and conjugated (direct) bilirubin are the two main pools of circulating bilirubin (Figure 1). Under healthy conditions, most of the total bilirubin in circulation is unconjugated bilirubin (UCB). Plasma bilirubin is maintained within specific limits by a dynamic equilibrium involving heme breakdown, bilirubin production, hepatic conjugation, intestinal metabolism, and partial enterohepatic cycling [18]. Because both excessive bilirubin accumulation and insufficient bilirubin synthesis may have cardiovascular effects, the relative effectiveness of these mechanisms is essential [9]. Unconjugated bilirubin is formed as part of the catabolism of red blood cells in the spleen through the actions of heme oxygenase. It is then released from the spleen into the blood, bound to albumin. Albumin-bound bilirubin is then transported to the liver for uptake and conjugation by UDP-glucuronosyltransferase 1A1 (UGT1A1) to increase its solubility for excretion. The canalicular membrane transporter multidrug resistance-related protein 2 (MRP2), also known as ABC-C2, is a member of the adenosine triphosphate (ATP)-binding cassette family. It mediates the excretion of bilirubin conjugates produced in hepatocytes to bile against a concentration gradient, where they are absorbed into mixed micelles containing bile acids, phospholipids, and cholesterol. They are transported to the intestine through the bile duct. In the colon, a small portion is deconjugated, mostly by bacterial enzymes (β-glucuronidase) in the gut microbiota. The resulting UCB can undergo intestinal reabsorption or be further processed in the intestinal tract [19]. The bacterial enzymes bilirubin reductase (BilR), from gut microorganisms including strains of *Clostridioides difficile*, *Clostridium ramosum*, *Clostridium perfringens*, and *Bacteroides fragilis*, reduce conjugated bilirubin to urobilinogen [20,21].

Urobilin is generated by gut microbiota as a byproduct of bilirubin catabolism (Figure 1). Bilirubin clearance commences in the liver, where UGT1A1 adds two glucuronic acid groups to the carboxyl tails of bilirubin, resulting in the formation of conjugated bilirubin [22]. The glucuronidation of bilirubin increases its solubility for excretion via the biliary system. Upon reaching the intestines, gut microbiota that express bilirubin reductase (BilR) remove the glucuronidation from conjugated bilirubin and reduce it to urobilinogen, which is subsequently oxidized to form urobilin [18]. BilR is predominantly expressed within the Firmicutes family and is present in the gut of nearly all healthy adults [20]. This is corroborated by previous research on bacteria in the genus Clostridium within the Firmicutes family, including *Clostridium difficile* [23], *Clostridium perfringens* [23], and *Clostridium ramosum* [24], which can reduce bilirubin to urobilinogen. Bacteria-produced urobilin is then either absorbed through the hepatic portal system or continues along the intestinal tract. Urobilin that remains in the intestines undergoes further metabolism to stercobilin by a currently unknown bacterial enzyme. Approximately 50% of the urobilin produced is absorbed and enters the systemic circulation [25]. Although the precise mechanism of absorption is unknown, urobilinogen is absorbed throughout the digestive tract [26]. Future studies are required to elucidate the mechanisms and regulatory processes governing urobilin absorption. Urobilin is ultimately excreted from systemic circulation into urine through the kidneys, giving urine its color. Understanding the pathways of urobilin production and absorption is essential for identifying potential therapeutic targets, such as UGT1A1 and BilR, to treat diseases associated with elevated urobilin levels.

### 2.2. Genetic Determinants of Elevated Bilirubin: Gilbert Syndrome and Cardiovascular Implications

Genetic variants affecting bilirubin metabolism further underscore its cardiovascular relevance. Gilbert syndrome (GS), primarily caused by promoter polymorphisms in UGT1A1 (commonly the UGT1A1*28 allele), results in reduced conjugation efficiency and mildly elevated unconjugated bilirubin levels [27,28]. It is important to note that the normal levels of bilirubin vary by age, sex, and race; however, individuals with GS consistently exhibit higher plasma bilirubin levels, ranging from 1 to 5 mg/dL (17.1 to 85.5 µmol/L) in males and females, compared to the general population with the range of bilirubin being as low as 0.2 mg/dL (3.4 µmol/L) in the adult European population and the upper range being as high as 2.2 mg/dL (37.6 µmol/L) in the African population [29,30,31]. Nevertheless, these patients generally remain asymptomatic, exhibit no signs of liver toxicity, and are safeguarded against harmful cardiometabolic dysfunction.

Epidemiological data suggest that GS confers cardiovascular protection, with lower rates of coronary artery disease, significantly reduced oxidative burden, and improved vascular profiles [32,33]. Mendelian randomization studies further support a causal relationship, showing that genetically elevated plasma bilirubin may reduce the risk of cardiovascular disease [28,34]. These findings suggest that moderate lifelong elevation of bilirubin may enhance cardiovascular resilience. Results from a more recent retrospective and prospective study indicated that mildly elevated serum bilirubin levels (especially in patients with the UGT1A1*28 allele) in patients with concurrent GS and hypercholesterolemia are protective against atherosclerotic cardiovascular disease [35]. In addition, several studies have demonstrated that GS patients are protected against metabolic diseases, including obesity and diabetes [36,37]. These findings have been corroborated by a study employing a humanized mouse model harboring the UGT1A1*28 polymorphism, which resulted in decreased body weight, diminished fasting blood glucose and insulin levels, and hepatic steatosis [38].

There are genetic forms of hyperbilirubinemia primarily associated with elevated conjugated bilirubin levels [18,39]. Dubin–Johnson syndrome results from mutations in the protein encoded by *ABC-C2* (formerly MRP2 [Multidrug Resistance Protein 2] or MOAT [Multispecific Organic Anion Transporter]), a member of the large ABC transporter family [40]. Alteration in this transporter leads to the accumulation of conjugated bilirubin. Rotor syndrome is another rare genetic disorder characterized by elevated conjugated bilirubin levels, arising from mutations in the Organic Anion-transporting Polypeptide 1B1 (OATP1B1) and OATP1B3, encoded by Solute Carrier Organic Anion Transporter Family Member 1B1 (SLCO1B1) and SLCO1B3, respectively [41,42]. Interestingly, neither patients with Dubin–Johnson nor Rotor syndrome have been reported to be protected against cardiovascular–metabolic diseases, suggesting that only elevated unconjugated bilirubin levels are protective.

## 3. Mechanisms by Which Elevated Bilirubin Levels Could Benefit the Heart

### 3.1. Antioxidant and Redox Regulation

**Figure 2:**
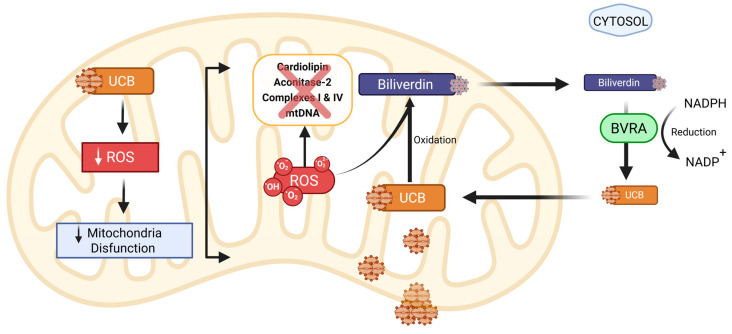
Antioxidant and redox regulatory mechanisms of bilirubin in mitochondria. Bilirubin enhances cardiac resilience by scavenging ROS, thereby preserving mitochondrial membrane integrity. Through redox cycling, bilirubin is oxidized to biliverdin, which is later reduced to bilirubin by BVRA. This redox recycling mechanism sustains intracellular antioxidant capacity and attenuates oxidative stress. Created in Biorender. Michael Adenawoola (2026). https://app.biorender.com/illustrations/69de5c1e7b8063a7fc09059e?slideId=84097cc6-2abe-4c1b-9eda-4bb8ed96b256 (accessed on 14 April 2026).

Bilirubin functions as a potent endogenous antioxidant, operating at multiple levels to limit oxidative injury, preserve membrane integrity, and modulate redox signaling (Figure 2). Three complementary mechanisms are central to bilirubin’s antioxidant properties: direct scavenging of reactive oxygen and nitrogen species (ROS/RNS), inhibition of enzymes that generate ROS, and protection against lipid peroxidation.

Unconjugated bilirubin is an efficient chain-breaking antioxidant that neutralizes a range of oxidants, including peroxyl radicals and reactive nitrogen species, thereby limiting oxidative damage to proteins, Deoxyribonucleic Acid (DNA), and lipids [43,44]. In the mitochondria, ROS-induced lipid peroxidation of polyunsaturated fatty acids leads to loss of membrane integrity and increased apoptosis. Cardiolipin, an anionic phospholipid of the inner mitochondrial membrane, is particularly vulnerable to lipid peroxidation [45]. Aconitase-2 is a mitochondrial enzyme with a ROS-sensitive (4Fe-4S) cluster, making it highly vulnerable to oxidative damage. Under stress or aging, it undergoes cluster loss, carbonylation, and degradation [46]. Electron transport chain complexes such as complex I and IV are both sources and targets of ROS, which increases electron leakage and mitochondrial DNA (mtDNA) damage, thereby causing mitochondrial dysfunction [47]. In cardiovascular tissues, the antioxidant property of bilirubin inhibits Low-Density Lipoprotein (LDL) oxidation and attenuates lipid peroxidation within cell membranes and in lipoprotein processes that underlie endothelial dysfunction and atherogenesis [10,48]. The antioxidant potency of bilirubin is thought to be amplified by the enzymatic interconversion between bilirubin and biliverdin. When bilirubin scavenges oxidants, it is converted back to biliverdin, which is then reduced to bilirubin by biliverdin reductase A (BVRA), regenerating the active antioxidant [44,49].

Small, steady-state bilirubin pools can provide long-lasting cytoprotection through this enzymatic “redox amplification.” Antioxidant recycling and cellular stress responses are integrated by BVRA’s dual roles as an enzyme and a signaling scaffold that connects redox buffering to kinase and transcriptional pathways [49,50]. Not only does bilirubin directly scavenge ROS, but it also inhibits ROS production. In a cell-free system consisting of the membrane and cytosolic fractions of neutrophils, bilirubin decreased superoxide production through the inhibition of Nicotinamide Adenine Dinucleotide Phosphate (NAD[P]H) oxidase activity [51]. In further studies in macrophages, bilirubin decreased both NAD(P)H oxidase activity and the expression of NAD(P)H oxidase subunits [52]. Lastly, studies in db/db diabetic mice demonstrated that bilirubin decreases the expression of NAD(P)H oxidase subunits in the kidney [53].

Mitochondria are both major sources and primary targets of ROS in cardiomyocytes. Recent mechanistic research shows that intracellular biliverdin/bilirubin metabolism is tightly coupled to mitochondrial function. First, the mitochondrial export of biliverdin via transporters (e.g., ATP-Binding Cassette Subfamily B Member 10 [ABCB10]) to the cytosol supports local conversion to bilirubin as BVRA is a cytosolic enzyme, thereby preserving mitochondrial redox homeostasis [54]. Second, BVRA and bilirubin modulate mitochondrial bioenergetics and dynamics: BVRA influences signaling pathways that regulate mitochondrial biogenesis and respiratory efficiency, whereas bilirubin reduces mitochondrial ROS production under stress, thereby limiting oxidative damage to mitochondrial proteins and membranes (Figure 2) [50]. Through these mechanisms, the HO–BVRA–bilirubin axis can blunt maladaptive ROS-driven signaling (for example, pathological opening of the mitochondrial permeability transition pore) and reduce downstream pro-inflammatory and pro-fibrotic cascades in the myocardium.

Bilirubin acts both as a radical scavenger and as a modulator of mitochondrial ROS production; therefore, modest, sustained elevations plausibly confer cardio protection by neutralizing ROS and increasing myocardial resilience to acute insults [55]. However, the protective window for bilirubin against ROS production in the heart depends on an optimal concentration; extremely low levels reduce antioxidant capacity, whereas very high levels can uncouple mitochondria, increasing ROS production.

### 3.2. Metabolic and Mitochondrial Effects

**Figure 3:**
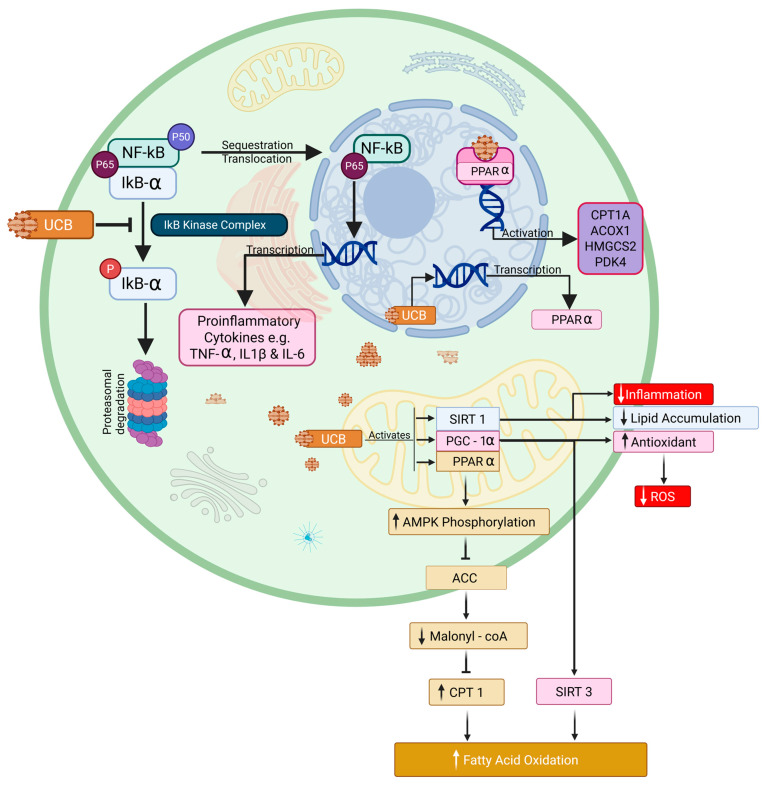
Proposed cardioprotective mechanisms of elevated bilirubin, including antioxidant activity, anti-inflammatory signaling, and fatty acid oxidation in cardioprotection. Bilirubin inhibits the NF-κB pathway by reducing the phosphorylation of IκB-α, inhibiting the IκB kinase (IKK) activity, stabilizing IκB-α, and preventing its degradation. NF-κB (p65/p50 heterodimer) is normally sequestered in the cytoplasm by binding to IκB-α. By stabilizing IκB-α, NF-κB (specifically the p65 subunit) remains sequestered in the cytoplasm, preventing its nuclear translocation and suppressing the transcription of pro-inflammatory cytokines, such as TNF-α, IL-1β, and IL-6. In summary, at normal-to-moderate levels, bilirubin stabilizes IκB-α to turn off NF-κB. However, very high bilirubin levels destabilize IκB-α, thereby activating NF-κB. In the mitochondria, Unconjugated Bilirubin (UCB) increases SIRT1 expression, thereby reducing hepatic lipid accumulation and inflammation. It also activates PGC-1α, which increases antioxidant production, thereby neutralizing Reactive Oxygen Species (ROS). Sirtuin 3 (SIRT3) is a target of PGC-1α and plays an important role in mitochondrial processes, including fatty acid oxidation. Bilirubin activates PPARα, increasing AMPK Phosphorylation. AMPK activates CPT 1 by inhibiting acetyl-CoA carboxylase (ACC), thereby lowering Malonyl-CoA levels, a molecule that normally inhibits CPT 1 activity. UCB binds to PPARα potentially via ligand-like activity or a direct mechanism to promote transcription of genes like HMGCS2, CPT1A, ACOX1, and PDK4 involved in fatty acid oxidation. Created in Biorender. Michael Adenawoola (2026). https://app.biorender.com/illustrations/69de5cb0aa7a20148e25e161?slideId=a0dbc4ca-e976-4aca-a539-72166bbe3eb7 (accessed on 14 April 2026).

Bilirubin enhances fatty acid oxidation (FAO) by upregulating metabolic regulators, such as peroxisome proliferator-activated receptor-α (PPARα), which activates transcriptional networks essential for increased FAO [56,57]. It also activates Peroxisome proliferator-activated receptor gamma coactivator 1-alpha (PGC-1α) and Sirtuin 1 (SIRT1) [58] (Figure 3). Further studies have demonstrated that chronic treatment with bilirubin nanoparticles remodels hepatic fat content by decreasing toxic ceramide levels and increasing phosphatidylethanolamine levels [59]. Phosphatidylethanolamine protects against the development of metabolic dysfunction-associated steatotic liver disease (MASLD) [60,61]. Studies in GS patients and mice also demonstrate that bilirubin activates the AMP-activated protein kinase (AMPK) pathway, which, as shown in Figure 2, increases FOA downstream [37,38]. Several studies have demonstrated that PPARα protects the heart. Treatment with PPARα agonists has been demonstrated to be protective in heart failure and myocardial infarction [62,63,64]. Cardiac PPARα levels are decreased in patients with heart failure with preserved ejection fraction (HFpEF), suggesting deficiencies in this pathway may contribute to the development and maintenance of this condition [65,66]. While bilirubin has been demonstrated to modulate PPARα in the liver and adipose, its effects on PPARα in the heart have yet to be tested. These types of studies will require measurements of cardiac metabolism following bilirubin treatment in normal as well as cardiomyocyte-specific knockouts. Nevertheless, there is a known interaction between hepatic PPARβ and the cardiovascular system, as hepatic knockout of PPARβ results in hypertension, impaired diastolic function, and increased vascular stiffness when maintained on a standard diet [67].

Bilirubin has been reported to have several effects on mitochondria. Early studies by Mustafa et al. reported that bilirubin has a biphasic effect on mitochondrial respiration in tissues such as the liver and heart, but not in the brain [68]. In more recent studies in adipose tissue, physiological concentrations of bilirubin (50 μM) have been reported to increase mitochondrial oxygen consumption rate and ATP production [69]. Mitochondrial integrity also appears to be maintained through bilirubin’s ability to suppress excessive mitochondrial reactive oxygen species (ROS) generation and preserve the activity of respiratory chain complexes. Through interactions with PGC-1α, a transcriptional co-activator and master regulator of mitochondrial biogenesis and energy metabolism, bilirubin supports mitochondrial turnover and maintains a healthy mitochondrial network [70]. Collectively, these metabolic effects could enable bilirubin to sustain cardiomyocyte ATP homeostasis, buffer against oxidative stress [18,71,72,73,74], and maintain energetic flexibility, which is particularly relevant in HFpEF, where mitochondrial dysfunction is a central pathogenic mechanism [75].

### 3.3. Anti-Inflammatory Mechanisms

Bilirubin has been reported to exert broad anti-inflammatory effects by suppressing pro-inflammatory signaling, modulating cytokine production, and regulating innate immune cell behavior. Several mechanistic axes have been described. Bilirubin, at physiological concentrations, can modulate inflammation both in vitro and in vivo [76,77,78]. A study by Li et al. demonstrates that physiological concentrations of bilirubin protect against inflammation, with mechanisms including inhibition of the Nuclear Factor kappa-light-chain-enhancer of activated B cell (NF-κB) signaling pathway and regulation of inflammasome activation (Figure 3) [79].

Suppression of NF-κB signaling is a well-established approach to limit inflammation. Unconjugated bilirubin decreases NF-κB activation in peritoneal macrophages by inhibiting its phosphorylation, and also reduces the secretion of classical pro-inflammatory cytokines like Tumor Necrosis Factor α (TNF-α), interleukin-1β (IL-1β), and interleukin-6 (IL-6). Both direct redox-dependent repression of upstream kinases and indirect activation of cytoprotective pathways that oppose NF-κB appear to contribute to this action [79]. Bilirubin is believed to have similar effects on cardiac macrophages and vascular endothelium. A modest increase in bilirubin in an in vivo study of apolipoprotein E-deficient (ApoE−/−) mice significantly reduced plasma glucose, total cholesterol, and atherosclerotic plaques, which indirectly reduces the risk of cardiovascular disease. It is suggested that bilirubin reduces plaques and endothelial damage by decreasing the expression of inflammatory mediators [76].

Bilirubin affects immune cell trafficking in the cardiovascular environment. It inhibits leukocyte recruitment to the heart and arterial wall by decreasing endothelial synthesis of chemoattractants and adhesion molecules (such as intercellular adhesion molecule-1 (ICAM-1), vascular cell adhesion molecule-1 (VCAM-1), and monocyte chemoattractant protein-1 (MCP-1)). By improving the lipid profile and modulating the expression of adhesion molecules, Lectin-like Oxidized Low-density Lipoprotein Receptor-1 (LOX-1), and Inducible Nitric Oxide Synthase (iNOS), mild hyperbilirubinemia can prevent the development of atherosclerosis and heart failure [80,81]. By suppressing NF-κB, limiting inflammatory cell influx, and promoting reparative macrophage phenotypes, bilirubin attenuates maladaptive inflammatory remodeling after metabolic or ischemic injury (Figure 3) [82]. These anti-inflammatory actions help explain epidemiologic associations between modestly elevated bilirubin and lower rates of atherosclerosis, myocardial remodeling, and heart failure [9,81]. However, because bilirubin is also an immunomodulator, chronically altered bilirubin homeostasis could have context-dependent effects (for example, impairing antimicrobial responses), so translational approaches must balance the benefits of inflammation suppression with host-defense considerations [83].

### 3.4. Endothelial and Vascular Effects

Bilirubin’s vascular actions extend beyond antioxidant buffering to include direct effects on endothelial nitric oxide signaling, endothelial integrity, and arterial wall structure, all of which are central to vascular aging and cardiovascular risk. Endothelial nitric oxide (NO) produced by endothelial nitric oxide synthase (eNOS) is a principal mediator of vasodilation and anti-atherogenic signaling. Oxidative stress lowers NO bioavailability by both quenching NO (forming peroxynitrite) and uncoupling eNOS enzymatic activity. Bilirubin preserves NO signaling by reducing oxidative inactivation of NO and by limiting eNOS uncoupling through its robust antioxidant effects [84]. Recent studies also implicate bilirubin in activating regulatory metabolic pathways (including SIRT1 and PPARα signaling) that positively modulate eNOS expression and phosphorylation, thereby enhancing NO generation under stress conditions [9,58]. In experimental models, modest increases in bilirubin correlate with improved endothelium-dependent vasodilation and restoration of NO-dependent vascular reactivity [84]. PPARα is recognized to regulate the expression of NOS in various tissues, including adipose tissue, as evidenced by the adipose-specific deletion of PPARα in male mice subjected to a high-fat diet, which resulted in significantly higher Nos2 expression compared to flox controls [85].

Endothelial dysfunction, characterized by impaired NO-mediated vasodilation, increased leukocyte adhesion, and prothrombotic signaling, is a crucial early step in atherogenesis. By scavenging ROS, downregulating adhesion molecule expression, and preserving mitochondrial function in endothelial cells, bilirubin decreases endothelial injury [81,84]. Clinical and population studies provide concordant evidence: individuals with Gilbert’s polymorphism exhibit better endothelial function and lower arterial stiffness markers than matched controls [84]. Because NO signaling and vascular compliance are tightly controlled, therapeutic strategies that modestly elevate bilirubin (e.g., transient HO-1 induction, biliverdin/bilirubin nanodelivery) must be titrated to preserve the protective window identified in epidemiological studies. Furthermore, the vascular benefits of bilirubin are interdependent with systemic metabolic and inflammatory states; consequently, interventions will likely be most effective when combined with approaches that address oxidative/inflammatory comorbidities [58,81].

### 3.5. Anti-Apoptotic and Cytoprotective Pathways

Bilirubin also exhibits robust cytoprotective effects in the myocardium, particularly by inhibiting apoptosis under pathological conditions. Experimental models demonstrate that bilirubin reduces ischemia–reperfusion (I/R) injury by limiting the opening of mitochondrial permeability transition pores, attenuating nicotinamide adenine dinucleotide phosphate oxidase (NOX) activation, and preserving membrane integrity [17]. These anti-apoptotic effects are complemented by bilirubin’s broader modulation of stress-response pathways, including enhancement of Nuclear Factor Erythroid 2-Related Factor 2 (Nrf2)-dependent antioxidant pathways, thereby improving cardiomyocyte survival during acute oxidative stress [86]. Moreover, bilirubin functions as a stress-response mediator, activating protective signaling cascades, including Extracellular signal-Regulated Kinases 1 and 2 (ERK1/2), in response to metabolic or inflammatory insults. By stabilizing mitochondrial function, suppressing cellular ROS, and promoting pro-survival signaling, bilirubin may confer a cardioprotective phenotype that mitigates the progression of myocardial injury and adverse remodeling [87]. Future studies are needed to investigate whether these effects also depend on bilirubin’s hormonal function. These properties highlight bilirubin as an endogenous cytoprotective molecule with therapeutic potential in conditions characterized by metabolic stress, oxidative injury, and mitochondrial dysfunction.

Endoplasmic reticulum (ER) stress occurs when misfolded proteins accumulate, triggering the unfolded protein response (UPR) to restore normal protein processing. ER stress is increased in metabolic diseases such as obesity and diabetes, as well as in inflammatory conditions and in neurological disorders. Bilirubin exerts divergent effects on ER stress. In neurons, high levels of bilirubin promote ER stress and UPR activation [88]. However, in vascular cells, increased bilirubin levels can reduce ER stress, especially in diabetes [89]. Bilirubin treatment was also demonstrated to decrease ER stress in chronic kidney disease [90]. However, the role of bilirubin on ER stress in the heart is not currently known.

## 4. Experimental and Preclinical Evidence

This section highlights translationally relevant studies from in vitro models, small- and large-animal studies, and genetic and pharmacological manipulations of bilirubin metabolism to study the role of bilirubin in safeguarding against myocardial ischemia–reperfusion (I/R) injury, pressure-overload hypertrophy, heart failure phenotypes—specifically Heart Failure with Preserved Ejection Fraction (HFpEF) versus Heart Failure with Reduced Ejection Fraction (HFrEF)—and models of metabolic disease.

### 4.1. Protection Against Ischemia–Reperfusion (I/R) Injury

The direct effects of bilirubin and associated pathway modifications on cardiomyocyte survival, redox balance, mitochondrial function, and endothelial phenotype have been examined using cellular models. Low-to-moderate exposure to bilirubin or biliverdin protects against oxidant-induced injury, limits lipid peroxidation, maintains mitochondrial respiration, and suppresses inflammatory signaling, according to several studies using primary cardiomyocytes, immortalized cardiomyoblast lines (such as H9C2), and cultured endothelial cells [54]. Mechanistically, these protective benefits include improved ATP maintenance in stress models, reduced ROS generation, and preserved electron transport chain complex function [54]. However, context and concentration are important. In a recent cell culture study, excessive bilirubin concentrations (>60 μM) were cytotoxic to cardiomyocytes, reducing cell viability and increasing markers of cell damage in a dose-dependent manner [91]. These results confirm the epidemiological association between bilirubin and cardiovascular events, highlighting a limited treatment window [92,93,94]. However, the literature suggests that increasing bilirubin to a mildly elevated level has beneficial effects without toxicity (further reviewed in [10]).

A study by Ai et al. using fluorophore-encapsulated bilirubin nanoparticles (BRNPs) demonstrated that BRNP treatment reduced infarct size and improved post-ischemic function in preclinical I/R models [17]. In mouse cardiac I/R models, administration of bilirubin nanoparticles significantly reduced infarct size, oxidative stress, inflammation, and enhanced functional recovery [17]. However, the effect of BRNPs on cardiac metabolism was not addressed in this study. Consistent with bilirubin-mediated antioxidant and anti-inflammatory processes, parallel strategies that pharmacologically (e.g., hemin pretreatment) or genetically stimulate HO-1 also protect against I/R damage across species [95]. These findings support the idea that reperfusion damage can be reduced by targeted administration or temporary activation of the HO–bilirubin axis.

### 4.2. Pressure Overload Hypertrophy

HO-1 induction attenuates hypertrophic remodeling and fibrosis in pressure-overload models, preserving systolic function [96]. Likewise, deletion of the HO-1 repressor, BTB and CNC homology 1 (BACH-1), causes a significant reduction in heart weight, tissue collagen content, levels of Atrial Natriuretic Peptide (ANP) and Brain Natriuretic Peptide (BNP), with reduced Left Ventricle (LV) dimensions and improved LV contractile function in a model of transverse aortic constriction (TAC) [97]. The results from these studies indicate that HO-1 induction limits pressure-induced ventricular remodeling and fibrosis; however, the specific role of bilirubin in this response remains unclear.

### 4.3. Heart Failure with Reduced Ejection Fraction (HFrEF) and Heart Failure with Preserved Ejection Fraction (HFpEF)

**Figure 4:**
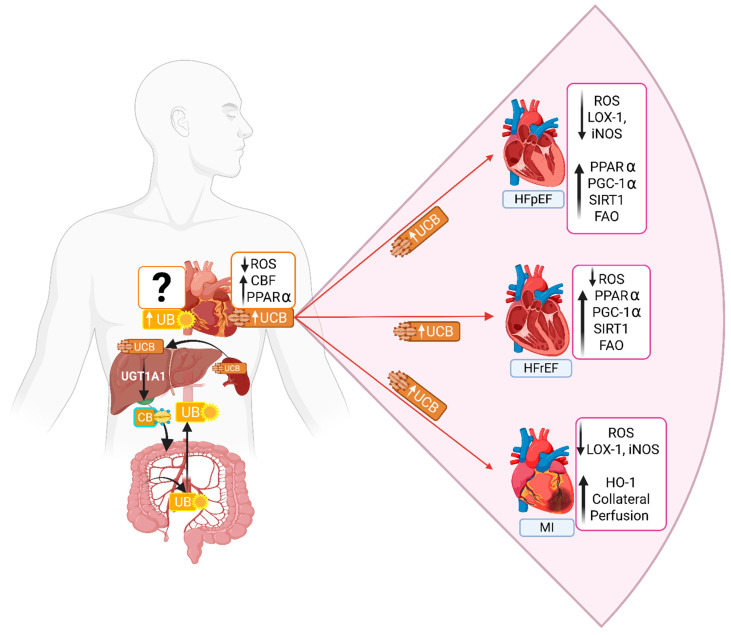
Integrated effects of elevated unconjugated bilirubin (UCB) on cardiac protection in heart failure with preserved ejection fraction (HFpEF), heart failure with reduced ejection fraction (HFrEF), and myocardial infarction (MI). UCB produced in the reticuloendothelial system of the spleen flows into the circulation. Increased circulating UCB is proposed to reduce reactive oxygen species (ROS) and LOX-1/iNOS expression while enhancing PPARα, PGC-1α, SIRT1, and fatty acid oxidation (FAO) in failing hearts. In myocardial infarction (MI), UCB additionally upregulates HO-1 and improves collateral perfusion. Hepatic UGT1A1 mediates bilirubin (CB) conjugation, regulating systemic UCB levels that exert cardioprotective effects across different cardiac pathologies. In the liver, UCB is conjugated through glucuronidation to form CB, which is further degraded to urobilin downstream of bacterial deconjugation. Urobilin (UB) is recirculated, with a portion excreted by the kidneys. The effect of urobilin on the heart under basal conditions and in pathological states such as HFpEF, HFrEF, and MI remains unknown. Created in Biorender. Michael Adenawoola (2026). https://app.biorender.com/illustrations/69de5cdc819bb6df16f487df?slideId=619cb059-bde7-423e-aac8-12edff01d1ae (accessed on 14 April 2026).

Heart failure (HF) is a syndrome characterized by symptoms such as breathlessness and fatigue, as well as clinical signs such as elevated jugular venous pressure and pulmonary congestion, which is caused by a structural and/or functional cardiac abnormality, resulting in reduced cardiac output and/or elevated intracardiac pressures at rest or during stress [98]. HF is divided into three major types: HF with reduced ejection fraction (HFrEF), in which ejection fraction is reduced by greater than 60% of normal; HF with preserved ejection fraction (HFpEF), which is defined as HF symptoms with impaired diastolic function exacerbated by aging, obesity or hypertension or a combination of the three factors; and HF with mildly reduced ejection fraction (HFmEF), which refers to HF with ejection fraction ranging from 41 to 49% of normal [98]. The function of bilirubin in HF may depend on ejection fraction status. The role of bilirubin in HFrEF remains controversial. Correlative studies in patients with HFrEF suggest that elevated bilirubin levels are detrimental [99]. This contrasts with studies indicating that higher plasma bilirubin levels correlate with better survival in patients with myocardial infarction [100]. One potential explanation for the differences in the role of bilirubin in HFrEF is the increased likelihood of liver complications that alter bilirubin metabolism, thereby raising plasma bilirubin levels beyond the protective range. Moderate increases in bilirubin may be beneficial in HFrEF when oxidative/inflammatory stress predominates (Figure 4) [95]. Bilirubin may have meaningful prognostic value in HF populations, though the direction and interpretation of associations vary by HF phenotype. In HFrEF, elevated unconjugated or total bilirubin levels generally reflect hepatic congestion and are independent predictors of worse outcomes [99]. Thus, bilirubin serves as a marker of disease severity in HFrEF, but may be a protective biomarker in HFpEF when within the normal or mildly elevated range [99]. Beyond heart failure, bilirubin also has prognostic relevance in ischemic stroke. Higher bilirubin at presentation is associated with smaller infarct size, reduced inflammation, and improved neurological outcomes after ischemic stroke [101]. In acute myocardial infarction (MI), moderate elevations in bilirubin correlate with reduced in-hospital complications, whereas extreme elevations may indicate hepatic stress or hemolysis [102]. These findings highlight bilirubin’s dual nature as both a protective marker and a disease indicator, depending on the context.

By contrast, HFpEF is a heterogeneous syndrome often driven by metabolic comorbidity, microvascular inflammation, and diastolic dysfunction; only recently have more translationally relevant HFpEF models (including metabolic syndrome-prone large-animal models) become available to test bilirubin-targeted strategies [103]. Experimental evidence indicates that systemic inflammation and oxidative stress are central drivers of coronary microvascular rarefaction and diastolic dysfunction in HFpEF, and that targeting these pathways can improve myocardial relaxation and vascular integrity [104]. In this context, bilirubin, as an endogenous antioxidant, has been proposed as a potential modulator of these pathophysiological processes [43]. In HFpEF, cohort studies have shown that conjugated, not unconjugated, bilirubin is associated with a poor short-term prognosis [105]. Little is known about the physiological signaling of conjugated bilirubin, but increased direct bilirubin is associated with adverse outcomes in HFpEF, while direct bilirubin is not. Given the established antioxidant properties of unconjugated bilirubin, it is plausible that differences in bilirubin fractions may reflect altered redox balance, although this mechanism has not been directly demonstrated [105]. However, direct, comprehensive comparisons of modulation of unconjugated versus conjugated bilirubin in HFpEF versus HFrEF remain limited and are an active area of preclinical research [103].

### 4.4. Genetic and Pharmacological Models Altering Bilirubin Metabolism

Genetic studies in rodents that modify UGT1A1 (conjugation) or HO-1 expression show that a lifelong mild increase in bilirubin [18], which mimics Gilbert syndrome, decreases oxidative stress markers [74] and increases vascular protection [38]. Conversely, excessive hyperbilirubinemia or significant interruptions of bilirubin clearance are harmful [106]. Mendelian randomization studies highlight the complexity and potential pleiotropy, yielding conflicting findings regarding the causal relationship between hyperbilirubinemia and CVD protection [106,107]. Pharmacologic approaches include HO-1 inducers (e.g., hemin), small-molecule BVRA modulators, and nanoparticle-delivered bilirubin. Hemin and other HO-1 inducers have consistently reduced organ injury in preclinical models, including cardiac ischemia–reperfusion (I/R) [95]. Bilirubin-loaded nanoparticles demonstrated robust cardioprotection in mouse ischemia–reperfusion (I/R) studies, with favorable biodistribution and controlled antioxidant delivery [17]. These strategies highlight two translational paths: (1) enhance endogenous bilirubin production through the induction of HO-1 and (2) the delivery of bilirubin directly in a targeted, controllable fashion (nanoparticles).

### 4.5. Urobilin and the Heart

Currently, urobilin has been shown to bind albumin [108] and has no other known functions or receptors. Urobilin has been primarily studied in humans and mice using non-targeted mass spectrometry and correlation analysis for nearly a century [39]. The earliest study linking urobilin to cardiovascular disease was published in 1930, where urobilin was detected in the urine of 88% of children with bedbound decompensated congestive heart failure and only 4% of control children [109]. This finding was supported by numerous other studies reporting elevated urinary urobilin/urobilinogen levels in patients with congestive heart failure [110,111], myocardial infarction [112,113], arteriosclerosis [114], and rheumatic endomyocarditis [115]. These studies support the potential use of urine urobilin as a biomarker for cardiovascular disease (further reviewed in [39]).

Recent human and preclinical rodent studies have focused on quantifying urobilin in plasma and correlating it with disease progression and severity. In a study involving over 3000 participants, urobilin was positively associated with the increased risk of cardiovascular disease mortality, stroke, and overall mortality [116]. Conversely, urobilin was significantly reduced in individuals with health-conscious dietary patterns [116]. Metabolomic profiling of three Swiss community-based cohorts revealed that plasma urobilin was positively associated with heart failure incidence (hazard ratio of 1.29 per standard deviation) and, in one cohort, negatively associated with baseline left ventricular ejection fraction [117]. Herreros-Cabello et al., using non-targeted metabolomics, found that urobilin was significantly elevated in the serum of patients with chronic chagasic cardiomyopathy compared with those with intermediate Chagas disease without cardiomyopathy, suggesting that urobilin may contribute to Chagas disease progression and cardiomyopathy [118]. In mice, urobilin was identified as the cecal metabolite most strongly associated with acute myocardial ischemia [119]. Nevertheless, additional research is required to ascertain whether urobilin functions solely as a biomarker for cardiovascular disease or also as a bioactive metabolite contributing to disease progression. Further investigations using preclinical rodent models are necessary to elucidate the mechanisms underlying the relationship between urobilin and cardiovascular disease.

In addition to being directly associated with cardiovascular disease, urobilin has also been correlated with recognized risk factors. In humans, urobilin was positively correlated with markers of adiposity and insulin resistance [120]. In lean patients, plasma urobilin levels were 9.57 μM (±4.50), whereas in patients with obesity, levels were 23.38 μM (±19.30) [120]. Notably, women with obesity exhibited higher urobilin levels, averaging 31.21 μM (±20.58) [120]. This finding was supported by Baek et al., who observed that urobilinogen was significantly higher in overweight participants with a high visceral fat area than in those with a low visceral fat area [121]. D-urobilinogen was positively correlated with LDL-cholesterol, oxidized LDL, and systolic and diastolic blood pressure [121]. In a study of over 700 patients with type 2 diabetes, plasma urobilin was positively associated with increased levels of blood glucose, BMI, triglycerides, smoking status, and all-cause mortality [122]. Conversely, plasma urobilin was negatively associated with HDL and anti-hypertensive treatment in the same study [122]. A recent untargeted metabolomics analysis found that urobilin was significantly higher in the plasma and urine of patients with liver cirrhosis compared to healthy control individuals [123]. Human studies supporting the association between urobilin and adiposity, insulin resistance, and liver disease have been corroborated by preclinical rodent models. Mice with diet-induced obesity have significantly higher urobilin in the cecal content compared to nonobese controls [124]. Utilizing a liver-specific RNAi against UGT1A1, Bates et al. significantly increased plasma bilirubin while decreasing urobilin in obese mice compared to obese controls [125]. These mice were protected against adiposity, glucose intolerance, and metabolic dysfunction-associated steatotic liver disease (MASLD) [125], highlighting the potential of targeting UGT1A1 to regulate urobilin levels as a treatment for cardiometabolic diseases. Collectively, these studies suggest that urobilin may contribute to obesity and insulin resistance, potentially leading to cardiovascular disease. However, the specific mechanisms require further investigation, as there are presently no studies examining the molecular mechanisms of urobilin in cardiometabolic dysfunction (Figure 4). Future studies are essential to elucidate the role of urobilin in both physiological and pathophysiological contexts.

## 5. Clinical and Translational Evidence

### 5.1. Epidemiological Associations

Large epidemiological studies consistently report an inverse relationship between circulating bilirubin concentrations and the risk of cardiovascular disease (CVD). Population-based cohorts such as the National Health and Nutrition Examination Survey (NHANES), the Framingham Offspring Study, and East Asian community registries demonstrate that individuals with higher physiologic bilirubin levels, typically in the upper tertile of normal, exhibit lower incidence of coronary artery disease (CAD), MI, stroke, and all-cause mortality [126,127]. These associations align with bilirubin’s potent antioxidant and anti-inflammatory properties, supporting its promising role as an endogenous cardioprotective factor. However, the role of bilirubin in cellular metabolism, acting through the PPARα nuclear receptor pathway [9], in bilirubin’s protective actions has yet to be directly tested.

Bilirubin has also been investigated as a biomarker across multiple cardiovascular conditions. In CAD, lower baseline bilirubin levels predict greater atherosclerotic burden, more rapid plaque progression, and a higher incidence of recurrent coronary events [32]. In HF, bilirubin levels correlate with disease severity, congestion, and right ventricular dysfunction, although elevations in advanced HF often reflect hepatic congestion rather than intrinsic protection [128]. Recent studies have also highlighted associations between bilirubin and arrhythmia risk, with lower bilirubin levels correlating with a higher prevalence of atrial fibrillation and ventricular arrhythmias, potentially due to impaired oxidative stress and electrophysiological stability [129]. Gilbert syndrome provides a natural human model to evaluate the protective role of moderate hyperbilirubinemia in CVD. Individuals with Gilbert syndrome exhibit a substantially lower incidence of CAD and metabolic syndrome, lower inflammatory markers, and improved endothelial function, collectively supporting a likely protective causal role of lifelong moderate bilirubin elevation [36,130,131].

### 5.2. Limitations of Clinical Evidence

The clinical interpretation of bilirubin is confounded by several significant constraints, despite robust relationships. First, a variety of circumstances, including liver illness, cholestasis, hemolysis, genetic variation, alcohol use, and drug usage (atazanavir, erythromycin, phenytoin, etc.), might affect plasma bilirubin levels. Isolating the causal effects of bilirubin on heart disease is difficult because these variables may confound correlations with outcomes. Second, individuals of different sexes, ages, and races exhibit distinct bilirubin dynamics. Due to hormonal and metabolic differences, men usually have greater bilirubin (0.72 ± 0.004 mg/dL) than women (0.52 ± 0.003 mg/dL); bilirubin decreases with age; and significant interethnic variance is caused by population-based variations in UGT1A1 allele frequency. Inadequate stratification of these factors may limit generalizability and bias risk assessments [132,133,134]. These discrepancies underscore the need for carefully planned interventions and long-term studies to determine whether altering bilirubin levels can significantly reduce cardiovascular events. Overall, more rigorous mechanistic and interventional trials are needed before bilirubin can be accepted as a therapeutic target or routinely used for cardiovascular risk stratification, despite the strong epidemiologic and clinical evidence.

## 6. Therapeutic Potential of Targeting Bilirubin Pathways

Interest in translational approaches to therapeutically modify bilirubin pathways has increased, driven by growing evidence linking bilirubin to cardioprotection. Current methods include the creation of synthetic bilirubin analogs and delivery systems, pharmacologic activation of endogenous bilirubin synthesis, and regulation of its conjugation and transport.

### 6.1. Pharmacological Modulation

HO1, BVRA, and UGT1A1 are enzymes in the bilirubin production pathway that have been proposed as therapeutic targets to increase unconjugated bilirubin levels (Figure 1). The rate-limiting enzyme in bilirubin production, HO-1, has been extensively studied as a potential therapeutic target. HO-1 inducers, including metalloporphyrins, natural polyphenols such as curcumin and resveratrol, pharmacologic agents such as bardoxolone methyl, and hemin, upregulate the HO-1 pathway, thereby increasing unconjugated bilirubin levels [135,136,137,138]. This increases intracellular bilirubin production and improves antioxidant and anti-inflammatory signaling. Induction of HO-1 in a diabetic mouse model using hemin has been shown to increase serum unconjugated bilirubin levels and restore endothelial function by increasing NO bioavailability via the Akt/eNOS/NO cascade [139]. These results indicate that HO-1 induction may be a viable therapeutic target for the clinical management of diabetic vasculopathy. In models of ischemia/reperfusion (I/R) injury, pressure overload, and metabolic dysfunction, HO-1 induction has been shown to confer protection. Another therapeutic approach, as shown in Figure 1, is to modify bilirubin conjugation and transport. Plasma pools of unconjugated bilirubin are regulated by hepatic UGT1A1. Although careful titration is necessary to prevent the risks associated with hyperbilirubinemia, pharmacologic downregulation or partial blockage of UGT1A1 raises physiological unconjugated bilirubin to cardioprotective levels. Transporters that regulate bilirubin uptake and efflux, such as OATP1B1, MRP2, and MRP3, have been identified as potential targets to maximize bilirubin bioavailability in tissues such as the heart and vasculature [140]. While UGT1A1 is an attractive target to increase unconjugated bilirubin levels, special consideration is needed when evaluating strategies to block it, especially in patients with cardiovascular disease who may be taking other drugs that require hepatic UGT1A1 for proper metabolism and elimination.

### 6.2. Novel Therapeutic Approaches

Recent innovations include engineered bilirubin nanoparticles designed to increase bilirubin solubility in aqueous solutions and improve delivery to cardiac tissue. Bilirubin-loaded nanoparticles can activate PPARα, exhibit potent antioxidant capacity, attenuate macrophage inflammation, and reduce myocardial injury in preclinical models. Synthetic bilirubin analogs with improved solubility and reduced toxicity are also under investigation [141]. Long-term modulation of bilirubin metabolism is now possible through gene-modulation approaches. Targeting UGT1A1 or HO-1 regulatory regions with CRISPR may result in a long-term increase in bilirubin that remains within physiological safety limits. Similarly, gene therapy strategies have demonstrated promise in enhancing endogenous cytoprotective mechanisms in cardiac and vascular tissues, such as viral delivery of biliverdin reductase or HO-1 [142]. Combined therapies are becoming more popular. Bilirubin-based treatments may enhance redox, metabolic, and endothelial benefits when combined with statins, SGLT2 inhibitors, or metabolic modulators (e.g., other PPARα agonists).

## 7. Limitations in the Potential for Bilirubin-Based Therapies

Although there is strong preclinical and epidemiological evidence that bilirubin may have cardioprotective effects, several important gaps in information and disagreements remain, making it challenging to translate these findings into practical treatment plans. Determining the exact thresholds at which bilirubin transitions from protective to detrimental is a significant source of ambiguity. Higher levels may suggest liver failure, cholestasis, or hemolysis, which can diminish the protective effects of bilirubin, even when moderate increases, as seen in Gilbert syndrome, are associated with lower cardiovascular risk [143,144]. One of the primary challenges remains the establishment of a reliable dosing regimen to optimize plasma unconjugated bilirubin levels.

The debate over causation versus correlation in human studies remains unresolved. Although many studies are unable to adequately account for variables including genetic background, metabolic health, alcohol use, or subclinical liver damage, observational data consistently show inverse relationships between plasma bilirubin levels and CVD outcomes. Mendelian randomization studies have yielded conflicting results, with some indicating a causal effect and others suggesting that bilirubin may function more as a biomarker of the systemic redox state than as an active mediator [34]. There is also substantial variation across comorbidities and populations. Differences in sex, age, and ethnicity affect bilirubin metabolism and its association with CVD outcomes, complicating the establishment of treatment objectives and universal reference ranges [126]. Additionally, the enzymatic pathways governing bilirubin synthesis and clearance may be altered by comorbidities such as diabetes, obesity, and chronic inflammation, thereby affecting bilirubin’s cardioprotective effects.

Between promising preclinical results and workable clinical therapies, a persistent translational gap remains. Studies on animals frequently employ models with increased HO-1 activity or supraphysiologic bilirubin concentrations, which are difficult to replicate in humans. Pharmacologic medicines that increase bilirubin carry the risk of off-target effects, while gene-based approaches require further testing for safety and efficacy. These difficulties underscore the need for integrative research that bridges systems biology, clinical trial design, and molecular pathways to fully understand the therapeutic potential of elevated bilirubin levels in cardiac diseases.

## 8. Future Perspectives

Personalized and precision medicine approaches have the potential to enhance future studies of bilirubin biology in CVD. Treatments targeting HO-1 or bilirubin metabolism may have distinct effects in individuals with genetic variants that elevate bilirubin, such as UGT1A1*28 carriers. According to Bansal et al., patients with hepatobiliary disease will most likely benefit from precision medicine, which, in this case, will stratify patients based on their genetic profile, hepatic function, and baseline bilirubin levels, and may help in customizing interventions and identifying which subgroups are most likely to benefit [145]. Another interesting approach is to incorporate bilirubin measures into models for CVD risk classification. Bilirubin may modify risk prediction for coronary artery disease, heart failure phenotypes, or vascular dysfunction when combined with established biomarkers, natriuretic peptides, hsCRP, or metabolic indicators, especially in younger people or those with low-grade chronic inflammation [127,146].

Emerging omics technologies provide opportunities to improve our understanding of bilirubin signaling. While single-cell transcriptomics and proteomics can identify cell-specific reactions to bilirubin in cardiomyocytes, endothelial cells, and macrophages, metabolomic profiling may reveal bilirubin-derived signaling lipids and redox intermediates [59]. These methods could reveal new receptors, interacting proteins, or redox-sensitive pathways that underlie bilirubin’s pleiotropic effects. New experimental models offer more opportunities for translation. Taken together, these prospective paths demonstrate bilirubin’s growing acceptance as both a biomarker and a potential therapeutic target for CVD. Translating bilirubin-centered insights into cardiovascular treatment will need strategic integration of genetics, systems biology, and next-generation modeling platforms.

## 9. Conclusions

Bilirubin has a significantly greater role in cardiovascular biology than previously thought, according to mounting evidence from molecular, preclinical, and clinical investigations. It is now known that bilirubin acts as a hormone that activates PPARα, in addition to being a powerful endogenous regulator of oxidative stress, inflammation, metabolism, endothelial function, and mitochondrial homeostasis. Previously, bilirubin was thought to be only a waste product of heme catabolism. However, studies have observed that bilirubin influences processes essential to the development of atherosclerosis, ischemia/reperfusion injury, metabolic dysfunction, and heart failure phenotypes, including HFpEF, through its dynamic cycling with biliverdin and its interactions with redox-sensitive pathways.

These mechanistic findings could lead to new treatment opportunities. Targeting this evolutionarily conserved system is appealing because it offers potential for pharmacological and genetic manipulation of HO-1, UGT1A1, and bilirubin transporters, as well as for the development of bilirubin nanoparticles and analogs. Bilirubin-based treatments may be effective when combined with established cardiometabolic therapies to reduce oxidative stress, preserve endothelial integrity and cardiac metabolism, and improve mitochondrial function in diseased myocardium. The field is moving quickly toward clinical practicality, despite remaining obstacles, such as establishing safe therapeutic thresholds, controlling interindividual variability, and bridging the gap between animal models and human translation.

A major paradigm shift in cardiovascular science is the reclassification of bilirubin from a metabolic byproduct to an active protector of cardiac function. Bilirubin has the potential to become a therapeutic target as precision medicine, multi-omics technologies, and sophisticated modeling platforms continue to emerge. Using this endogenous chemical could eventually enable new options for treating and preventing cardiovascular disease, especially in complex cases like HFpEF, where effective treatments remain limited.

## Data Availability

No new data were created for this manuscript.

## Abbreviations

The following abbreviations are used in this manuscript:

ABCB10	ATP-binding Cassette Subfamily B Member 10
ABC-C2	ATP-binding Cassette Subfamily C Member 2
ACOX1	Acyl-CoA Oxidase 1
Akt	Ak Strain Transforming or Protein Kinase B
AMPK	AMP-activated Protein Kinase
ANP	Atrial Natriuretic Peptide
ApoE−/−	Apolipoprotein E-deficient
ATP	Adenosine Triphosphate
BACH-1	BTB and CNC Homology 1
BilR	Bilirubin Reductase
BNP	Brain Natriuretic Peptide
BRNP	Bilirubin Nanoparticles
BVRA	Biliverdin Reductase A
CAD	Coronary Artery Disease
CB	Conjugated Bilirubin
CRISPR	Clustered Regularly Interspaced Short Palindromic Repeats
CPT1A	Carnitine Palmitoyltransferase 1A
CVD	Cardiovascular Disease
DNA	Deoxyribonucleic Acid
eNOS	Endothelial Nitric Oxide Synthase
ERK1/2	Extracellular Signal-regulated Kinases 1 and 2
FAO	Fatty Acid Oxidation
GS	Gilbert’s Syndrome
HF	Heart Failure
HFpEF	Heart Failure with Preserved Ejection Fraction
HFrEF	Heart Failure with Reduced Ejection Fraction
HMGCS2	3-Hydroxy-3-Methylglutaryl-CoA Synthase 2
HO-1	Heme Oxygenase-1
hsCRP	High-sensitivity C-reactive Protein
I/R	Ischemia–reperfusion
ICAM-1	Intercellular Adhesion Molecule-1
IL-1β	Interleukin-1β
IL-6	Interleukin-6
iNOS	Inducible Nitric Oxide Synthase
LDL	Low-density Lipoprotein
LOX-1	Lectin-like Oxidized Low-density Lipoprotein Receptor-1
LV	Left Ventricle
MASLD	Metabolic Dysfunction-associated Steatotic Liver Disease
MCP-1	Monocyte Chemoattractant Protein-1
MI	Myocardial Infarction
MOAT	Multispecific Organic Anion Transporter
MRP2	Multidrug Resistance-associated Protein 2
MRP3	Multidrug Resistance-associated Protein 3
NAD(P)H	Nicotinamide Adenine Dinucleotide Phosphate
NF-kB	Nuclear Factor Kappa-light-chain-enhancer of Activated B cells
NHANES	National Health and Nutrition Examination Survey
NO	Nitric Oxide
NOX	Nicotinamide Adenine Dinucleotide Phosphate Oxidase
Nrf2	Nuclear Factor Erythroid 2-related Factor 2
OATP1B1	Organic Anion-transporting Polypeptide 1B1
OATP1B3	Organic Anion-transporting Polypeptide 1B3
PDK4	Pyruvate Dehydrogenase Kinase 4
PGC-1α	Peroxisome Proliferator-activated receptor gamma coactivator 1-alpha
PPARα	Peroxisome Proliferator-activated Receptor-α
RNAi	Ribonucleic Acid Interference
RNS	Reactive Nitrogen Species
ROS	Reactive Oxygen Species
SGLT2	Sodium Glucose Cotransporter 2.
SIRT1	Sirtuin 1
SLCO1B1	Solute Carrier Organic Anion Transporter Family Member 1B1
SLCO1B3	Solute Carrier Organic Anion Transporter Family Member 1B3
TAC	Transverse Aortic Constriction
TNF-α	Tumor Necrosis Factor Alpha
UB	Urobilin
UCB	Unconjugated Bilirubin
UGT1A1	UDP-glucuronosyltransferase 1A1
VCAM-1	Vascular Cell Adhesion Molecule-1
